# Assessing the Competence of Artificial Intelligence Programs in Pediatric Ophthalmology and Strabismus and Comparing their Relative Advantages

**DOI:** 10.22336/rjo.2023.62

**Published:** 2023

**Authors:** Eyupcan Sensoy, Mehmet Citirik

**Affiliations:** * Department of Ophthalmology, Ankara Etlik City Hospital, Ankara, Turkey

**Keywords:** artificial intelligence, Bing, Bard, ChatGPT, pediatric ophthalmology

## Abstract

**Objective:** The aim of the study was to determine the knowledge levels of ChatGPT, Bing, and Bard artificial intelligence programs produced by three different manufacturers regarding pediatric ophthalmology and strabismus and to compare their strengths and weaknesses.

**Methods:** Forty-four questions testing the knowledge levels of pediatric ophthalmology and strabismus were asked in ChatGPT, Bing, and Bard artificial intelligence programs. Questions were grouped as correct or incorrect. The accuracy rates were statistically compared.

**Results:** ChatGPT chatbot gave 59.1% correct answers, Bing chatbot gave 70.5% correct answers, and Bard chatbot gave 72.7% correct answers to the questions asked. No significant difference was observed between the rates of correct answers to the questions in all 3 artificial intelligence programs (p=0.343, Pearson’s chi-square test).

**Conclusion:** Although information about pediatric ophthalmology and strabismus can be accessed using current artificial intelligence programs, the answers given may not always be accurate. Care should always be taken when evaluating this information.

## Introduction

With the development of technology, artificial intelligence applications, which are a sub-branch of computer science, have emerged and made a wide variety of contributions to the use of all fields of medicine. Artificial intelligence is a system based on the human mindset and developed to give similar reactions [**1**]. Although it is thought to have been mentioned for the first time during a conference in 1956, the first studies were delayed until the early 1970s [**2**]. These applications contain a wide variety of useful applications such as interpreting images, perceiving spoken words, and producing solutions to emerging problems [**3**]. Since 2015, the interest in artificial intelligence in ophthalmology applications has increased, and there have been various developments in deep learning applications [**4**]. Systems that recognize a wide variety of images such as fundus photographs and optical coherence tomography have been developed and useful results have been obtained in the detection of diseases [**5**,**6**]. Pediatric ophthalmology and strabismus are some of the important fields in which artificial intelligence applications find their place [**7**,**8**]. Artificial intelligence is also used to provide fast and reliable access to correct information by using chatbots [**9**]. Chat Generative Pre-Trained Transformer (ChatGPT) developed by OpenAI, Bing developed by Microsoft, and Bard artificial intelligence chatbots developed by Google AI are important examples of this group.

The aim of this study is to examine artificial intelligence chatbots such as ChatGPT, Bing, and Bard, which have entered our lives recently and whose provided information is often accepted as accurate, to evaluate the accuracy levels of their answers to multiple choice questions on topics related to pediatric ophthalmology and strabismus and to discuss the existence of advantages/disadvantages to each other.

## Methods

Forty-four questions containing information about pediatric ophthalmology and strabismus were taken from the study questions of the American Academy and Ophthalmology 2022-2023 Basic and Clinical Science Course Pediatric Ophthalmology and Strabismus book [**10**]. 27 of these 44 questions tested the knowledge levels of pediatric ophthalmology, and 17 questions tested the knowledge levels of strabismus. The question was asked to three artificial intelligence chatbots on June 27, 2023. ChatGPT GPT-3.5 (OpenAI; San Francisco, CA), Bing (Microsoft, Redmond, WA), and Bard (by Google AI) artificial intelligence chatbots. “I’m going to ask you multiple-choice questions. Please tell me the correct answer option” command was given. All questions were asked for 3 artificial intelligence programs separately and individually. The chat session was restarted after each question to avoid the retention effect of artificial intelligence programs. The answers given by the chatbots to the questions were compared with the answer keys and grouped as correct or incorrect. In addition, correct and incorrect answers were classified according to their level of knowledge.

Statistical Package for the Social Sciences version 23 (SPSS Inc., Chicago, IL, USA) was used for statistical analysis of the data. Percentages were used to evaluate descriptive data, and Pearson’s chi-square and Yates’ chi-square tests were used to compare nominal independent data. A p<0.05 value was considered significant when evaluating the statistical analyses.

## Results

ChatGPT gave correct answers to 26 (59.1%) of the 44 questions and incorrect answers to 18 (40.9%). Of the 26 correctly answered questions, 7 (26.9 %) measured information about strabismus, and 19 (73.1%) measured information about pediatric ophthalmology. While 10 (55.6%) of the 18 wrongly answered questions were testing information about strabismus, 8 (44.1%) were measuring information about pediatric ophthalmology.

Bing gave correct answers to 31 (70.5%) of the 44 questions and incorrect answers to 13 (29.5%) questions. While 10 (32.3%) of the 31 questions Bing answered correctly tested the knowledge level about strabismus, 21 (67.7%) tested the knowledge regarding pediatric ophthalmology. Seven (53.8%) of the 13 questions that were answered incorrectly measured information about strabismus, and 6 (46.2%) were testing information about pediatric ophthalmology.

Bard gave correct answers to 32 (72.7%) of the questions asked and incorrectly to 12 (27.3%) of the questions asked. Of the 32 questions Bard answered correctly, 11 (34.4%) measured information about strabismus, while 21 (65.6%) measured information about pediatric ophthalmology. 6 (50%) of the 12 questions with incorrect answers were testing information about strabismus, and 6 (50%) were testing information about pediatric ophthalmology.

All artificial intelligence chatbots provided the same answers to 26 (59.1%) questions. Of the questions with common answers, 20 (76.9%) were answered correctly and 6 (23.1%) incorrectly. Of the 20 questions answered correctly, 5 (25%) were testing information about strabismus, while 15 (75%) were testing information about pediatric ophthalmology. While 4 (66.7%) of the 6 wrongly answered questions measured information about strabismus, 2 (33.3%) measured information about pediatric ophthalmology (**Table 1**).

**Table 1:** The success of artificial intelligence chatbots on questions related to pediatric ophthalmology and strabismus

Answers (n)	ChatGPT	Bing	Bard
Correct	26 (59.1%)	31 (70.5%)	32 (72.7%)
Pediatric Ophthalmology/Strabismus	19/7	21/10	21/11
Incorrect	18 (40.9%)	13 (29.5%)	12 (27.3%)
Pediatric Ophthalmology/Strabismus	8/10	6/7	6/6
Same Answers (n)	26 (59.1%)		
Correct	20 (76.9%)		
Pediatric Ophthalmology/Strabismus	15/5		
Incorrect	6 (23.1%)		
Pediatric Ophthalmology/Strabismus	2/4		

No significant difference was observed in the correct and incorrect response rates for any of the three artificial intelligence programs (p=0.343, Pearson’s chi-square test). There was no significant difference between the rates of correct and incorrect answers to questions between the ChatGPT and Bing chatbots (p=0.372, Yates chi-square). No significant difference was observed between the rates of correct and incorrect answers to questions between Bing and Bard chatbots (p=1.0, Yates chi-square). No significant difference was observed between the rates of correct and incorrect answers to questions between ChatGPT and Bard chatbots (p=0.261, Yates chi-square test).

## Discussion

Artificial intelligence is a new species that constantly renews itself and continues to have an effective say in the world we live in. Artificial intelligence programs produced in the past were deep learning models produced with the aim of learning patterns [**11**]. Large Language Models (LLM) are computer programs that can understand users’ expressions, summarize them, identify various possibilities, and draw conclusions from them [**12**]. ChatGPT, Bing, and Bard chatbots are artificial intelligence applications developed based on LLM.

Developed based on LLM, ChatGPT is an artificial intelligence chatbot with 175 billion parameters and is trained to respond appropriately to human thought. The fact that it has been trained with such a wide range of parameters has made it stand out among the existing artificial intelligence programs [**11**]. Thanks to its various advantages such as being able to make customizable learning plans, having a language translation feature, assisting research, and fast access to information, it has enabled various uses in medical education [**9**]. It also presents a wide range of benefits, such as obtaining information about a wide variety of diseases, researching differential diagnoses, and providing information about treatment. Considering these benefits, it can have beneficial effects on a wide range of groups from medical students to health professionals [**13**]. In addition, its features, such as the ability to scan the literature, find text summaries, and analyze data prevent the medical researcher from getting lost in a large amount of information on the internet, and mediate access to information quickly and reliably [**14**]. Bing and Bard artificial intelligence programs were put into use this year. These are currently artificial intelligence programs with ChatGPT-like features. Bing chatbot is an artificial intelligence program that tends to generate short and direct answers as a result of internet-based interactions [**15**]. On the other hand, the Bard chatbot is an artificial intelligence program that has the ability to understand what is being told and to create a coherent new storyline about it. The fact that these artificial intelligence programs provide an advantage in rapid access to current and accurate information suggests that they can be used in the diagnosis and treatment of pediatric ophthalmology and strabismus diseases and that they can be a safe resource that can be used for fast and correct decision-making, considering the intense polyclinic conditions and the difficulties in examining children, as well as the different sensitivities of families. However, these programs have some disadvantages and raise many questions regarding their reliability. Examples of these disadvantages are ChatGPT’s coverage of 2021 and earlier data, and artificial intelligence chatbots being able to access only freely accessible information on the Internet (some articles cannot be accessed by these programs due to paid access) [**11**,**16**]. Medicine is a science that constantly develops and renews itself. Disruption in access to up-to-date information can lead to errors in diagnosis and treatment and difficulties in the examination and treatment of pediatric patients can complicate misdirection. Considering the wide variety of advantages of artificial intelligence chatbots, although they are encouraging for clinical applications, their reliability is confusing when considering their disadvantages, and it is important to measure their performance on these issues [**17**,**18**]. In this research, we aimed to test the performance of artificial intelligence chatbots on these subjects by measuring the correct answer rates they gave to multiple-choice questions about pediatric ophthalmology and strabismus.

In recent years, many studies have examined correct answer rates to evaluate medical questions. One of these studies was conducted in China, in which the answers to 12,723 questions were examined. Researchers stated that at the end of that study, the accuracy rate was 36.7% [**19**]. Later, as a result of the developments in artificial intelligence, the use of LLM-based artificial intelligence chatbots that can understand, analyze, and respond to more complex sentences has come to the fore. In a study measuring the success of the ChatGPT artificial intelligence program in correctly answering the questions in the USMLE, it was stated that there was a correct answer rate of over 50% [**20**]. It was also stated that a correct answer rate of 46% was found in a study conducted with the ChatGPT chatbot to determine the level of knowledge about ophthalmology [**21**]. In another recent study, it was found that ChatGPT and Bing chatbots evaluated the proficiency levels of their ophthalmology knowledge and answered correctly at a rate of 58.8% and 71.2%, respectively [**16**]. Similar to the literature, in our own study, we found that the lowest correct answer rate, 59,1%, was in ChatGPT. However, this rate was above 70% in Bing and Bard artificial intelligence chatbots, which were put into use in 2023. Although there was no significant difference between the correct answer rates of these 3 artificial intelligence programs, the correct answer rates of Bing and Bard chatbots in the fields of pediatric ophthalmology and strabismus were higher than ChatGPT. The fact that these artificial intelligence programs are more up-to-date may be related to ChatGPT’s limited access to data from 2022 and beyond. The fact that the Bard artificial intelligence program has a higher correct answer rate than the Bing artificial intelligence program may be related to the Bard chatbot’s ability to establish the context between words and analyze the plot better.

The limitations of the study are that artificial intelligence programs have barriers to accessing all the information in the literature, ChatGPT’s use of 2021 and previous information, and the low number of questions.

## Conclusion

To our knowledge, this study is the first to examine and compare the performance of artificial intelligence chatbots, launched by 3 different LLM-based manufacturers, on multiple choice questions related to pediatric ophthalmology and strabismus. Although the superiority of the more up-to-date Bing and Bard artificial intelligence programs over ChatGPT was not found to be statistically significant, the use of these programs in accessing accurate information can be considered a priority. However, given the inaccuracy of information and the limited possibility of accessing up-to-date information, care should always be taken in the evaluation of information.

**Conflict of Interest**

The authors declare that they have no conflict of interest.

**Acknowledgments**

None. 

**Sources of Funding**

None. 

**Disclosures**

None.
